# Development of a New Hydrogel Anion Exchange Membrane for Swine Wastewater Treatment

**DOI:** 10.3390/membranes12100984

**Published:** 2022-10-10

**Authors:** Peter Babiak, Geoff Schaffer-Harris, Mami Kainuma, Viacheslav Fedorovich, Igor Goryanin

**Affiliations:** 1 Biological Systems Unit, Okinawa Institute of Science and Technology Graduate University, Okinawa 904-0495, Japan; 2 School of Informatics, University of Edinburgh, Edinburgh EH8 9YL, UK

**Keywords:** anion exchange membrane (AEM), electrodialysis, bioelectrochemical denitrification system (BEDS), swine wastewater treatment, nitrogen removal, phosphate recovery, porous ceramic support, permselectivity, positively charged monomers

## Abstract

We developed a proprietary anion exchange membrane (AEM) for wastewater treatment as an alternative to commercial products. Following the successful development of a hydrogel cation exchange membrane on a porous ceramic support, we used the same approach to fabricate an AEM. Different positively charged monomers and conditions were tested, and all AEMs were tested for nitrate and phosphate anion removal from buffers by electrodialysis. The best AEM was tested further with real swine wastewater for phosphate removal by electrodialysis and nitrate removal in a bioelectrochemical denitrification system (BEDS). Our new AEM showed better phosphate removal compared with a commercial membrane; however, due to its low permselectivity, the migration of cations was detected while operating a two-chambered biocathode BEDS in which the membrane was utilized as a separator. After improving the permselectivity of the membrane, the performance of our proprietary AEM was comparable to that of a commercial membrane. Because of the usage of a porous ceramic support, our AEM is self-supporting, sturdy, and easy to attach to various frames, which makes the membrane better suited for harsh and corrosive environments, such as swine and other animal farms and domestic wastewater.

## 1. Introduction

Nitrogen and phosphorus in wastewater (WW) have major impacts on the environment and human health when left untreated [1]. Current WW treatment technology predominantly uses aeration with activated sludge method to remove organic contaminants, which are measured by the biological oxygen demand (BOD) or chemical oxygen demand (COD) of the WW [2]. However, this technology does not remove nitrate or phosphate effectively, and without proper subsequent treatment, these compounds remain in WW.

Phosphate is a necessary component of all fertilizers. Since phosphate cannot be synthesized like ammonia or nitrate, it must be mined, making it a very scarce resource. A number of studies have been conducted on the recovery of phosphate from WW [3,4]. Swine WW is a relatively rich source of phosphate (80–100 mg/L after aeration). Removal and recovery of phosphate from swine WW is necessary not only for ecological reasons (underground water pollution, eutrophication), legislative reasons (limits on WW discharge), and public image (eco-hype) but also for economic reasons [5]. 

Electrochemical methods, such as electrocoagulation or electrooxidation, are also used for swine WW treatment [6]. A report on phosphate separation from WW using cation exchange membranes (CEM), anion exchange membranes (AEM), and monovalent selective ion exchange membranes showed 93% recovery of phosphate [7]; however, the study was conducted on anaerobic waste (difficult to use for swine WW) and used a complexed system requiring expensive membranes. A simpler reactor configuration for the recovery of phosphate from domestic WW was described in a previous study [8], although this system had only been tested on synthetic WW. Furthermore, both of these reactor designs required brine for their function. 

All of these previously reported approaches used commercially available membranes that had mainly been developed for high-current/voltage applications. For WW treatment, membranes should be low cost, low biofouling, and have mechanical resistance. Currently, a membrane meeting all these requirements is difficult to find on the market. For this reason, we developed an easy to make, self-supporting hydrogel AEM on a porous ceramic carrier. Porous ceramic was chosen because of its long history in water treatment processes, such as filtration [9] and WW aeration, and its availability on the market. 

We tested our AEM in the post-treatment of swine WW after the activated sludge method (aeration) with the goal of meeting environmental discharge limits of phosphate and nitrate. We first tested for phosphate removal and concentration by electrodialysis; concentrated phosphate can be precipitated further as a form of struvite [10]. The membrane was then tested as a separator in a bioelectrochemical denitrification system (BEDS) developed in our laboratory [11].

## 2. Materials and Methods

### 2.1. Chemicals, Materials, and Equipment

All chemicals used were commercially available from Sigma-Aldrich (St. Louis, MO, USA). Porous ceramic plates of 40% porosity and 97 × 75 × 2 mm in size were purchased from Nikko Company (Ishikawa, Japan). Comparative tests were carried out with commercial membrane AMI-7001 (Membranes International Inc., Ringwood, NJ, USA). A Squidstat Prime potentiostat (Admiral Instruments, Tempe, AZ, USA) was used for electrochemical experiments. Masterflex L/S Digital Process Pumps (Cole-Parmer, Niles, IL, USA) were used for pumping solutions. Aerated swine WW was collected from an oxidation ditch at Okinawa Prefecture Livestock and Grassland Research Center, Okinawa, Japan.

### 2.2. Chemical Analysis

Concentrations of COD (TNT25), nitrate (TNT835), phosphate (TNT845), and ammonia (TNT833) were analyzed using HACH test kits and measured using a DR 3900 spectrophotometer (HACH Company, Loveland, CO, USA). The Instrument and Analysis Section at the Okinawa Institute of Science and Technology Graduate University (OIST), Japan assisted with ion chromatography measurements of chloride and sulfate concentrations.

### 2.3. Monomer Selection

**Figure 1:**
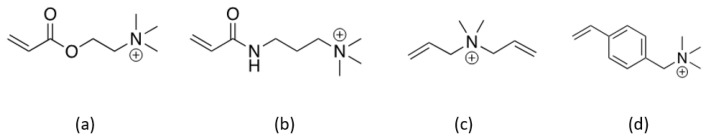
List of charged monomers used: (**a**) [2-(acryloyloxy)ethyl]trimethylammonium; (**b**) (3-acrylamidopropyl)trimethylammonium; (**c**) diallyldimethylammonium; (**d**) (vinylbenzyl)trimethylammonium. All monomers have Cl^−^ as a counterion.

We first tested four different positively charged monomers (Figure 1). Ten milliliters of 1 M solutions of each monomer in deionized water were prepared in 50 mL Falcon tubes. Three different concentrations (2, 4, and 6 mol%) of the crosslinker (CL) *N*-(acryloylamino-methyl)-acrylamide were added to each monomer and stirred at room temperature until the CL had solubilized. After CL solubilization, the solutions were cooled to 4 °C and ammonium persulfate (APS), a radical initiator, was added to a final concentration of 2 mol%, and the solutions were stirred at 4 °C until the radical initiator had solubilized. All solutions were heated to 60 °C for 24 h for polymerization and then cooled to room temperature for examination. Samples which failed to polymerize to gel were discarded. Samples which polymerized to a strong gel were cooled to 4 °C before the addition of 10 mL of 2 M acrylamide solution supplemented with 0.3% CL and 2% APS. Samples were stored at 4 °C for 24 h before being examined for acrylamide swelling.

### 2.4. Membrane Preparation Process

A polymerization reactor was constructed using 8-mm-thick acrylic glass. The rectangular reactor had dimensions of 50 (height) by 100 × 80 mm (area). The lid of the reactor had two taps for gas exchange.

The first polymer solution was generated as follows: 25 mL of 4 M diallyldimethylammonium chloride (monomer) solution and 308 mg for 2 mol%, 616 mg for 4 mol%, or 924 for 6 mol% of *N,N′*-methylenebisacrylamide (CL) were solubilized in 75 mL of deionized water. The solution was cooled to 4 °C and sparged with nitrogen for 15 min. Then, 456 mg (2 mol%) of APS was added and solubilized. Air in the porous ceramic was exchanged to nitrogen in the exicator by applying a vacuum and then filling with nitrogen. The procedure was repeated three times. The ceramic was then cooled to 4 °C and dipped into the chilled monomer solution containing the CL and APS. Vacuum was applied for 1 h to fill all pores with the solution. The reaction was kept at 4 °C. The vacuum was replaced by a nitrogen atmosphere, and the ceramic was heated to 60 °C for 24 h before being cooled to room temperature. Excess gel was scraped off from the ceramic. The ceramic was impregnated with the first polymer and cooled to 4 °C. The second polymer solution was prepared by solubilizing 14.2 g of acrylamide (monomer) and 92.4 mg of the CL in 100 mL of deionized water, chilled to 4 °C, and then sparged with nitrogen for 15 min. Then, 912 mg of APS was added and solubilized. The ceramic with the first polymer was dipped into the chilled second polymer solution for 24 h at 4 °C before being heated to 60 °C for 24 h and cooled to room temperature. The membrane was ready for use once excess gel had been scraped off.

To produce an AEM with improved permselectivity, the first polymer solution was prepared as follows: 50 mL of 4 M diallyldimethylammonium chloride solution and 616 mg (2 mol%) of *N,N′*-methylenebisacrylamide were solubilized in 50 mL of deionized water. The solution was cooled to 4 °C and sparged with nitrogen for 15 min. Then, 912 mg (2 mol%) of APS was added and solubilized. The remaining procedure was the same as the method described above.

### 2.5. Electrodialysis

A two-chambered electrochemical cell was constructed in the form of a hollow cylinder with one closed side and an open side with a flange and rubber seal. The volume of each chamber was 50 mL and was separated by the AEM. Each chamber had non-porous carbon fiber cloths (50 mm in diameter) as electrodes (Sohim, Svetlogorsk, BY) and two ports with nipples for electrolyte solution filling. The nipples had silicone tubing attached for better mixing. The AEM was pressed between rubber rings located on each flange. Each cell was connected to a potentiostat for chronoamperometry. 

**Figure 2:**
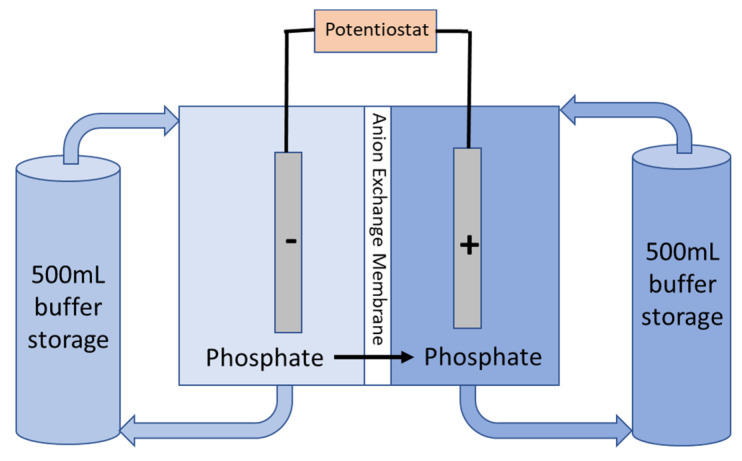
Schematic diagram of the electrodialysis experiment.

Electrodialysis experiments were conducted at three different potentials (0.5, 1.0, and 1.5 V) and using 200 mM phosphate buffer (pH 7). Both chambers were filled and circulated with 500 mL of the buffer using a peristaltic pump at a flow rate of 10 mL/min for 24 h (Figure 2). Results from chronoamperometry provided difference curves which represented the dynamics of the phosphate ion current with respect to time. Initial and final phosphate concentrations in each chamber were measured using HACH kits. The best AEM was determined by the highest phosphate ion transfer.

Electrodialysis experiments using aerated swine WW were carried out using the best AEM, at 1.5 and 2 V, and with the same conditions as the phosphate buffer experiment. Initial and final phosphate and nitrate concentrations were measured and compared with the commercial AEM as a benchmark. Membrane anion selectivity was evaluated by calculating (as mol%) the removal of the most abundant anions (phosphate, chloride, sulphate, and nitrate) from the aerated swine WW.

### 2.6. Electrotrophic Denitrification System

**Figure 3:**
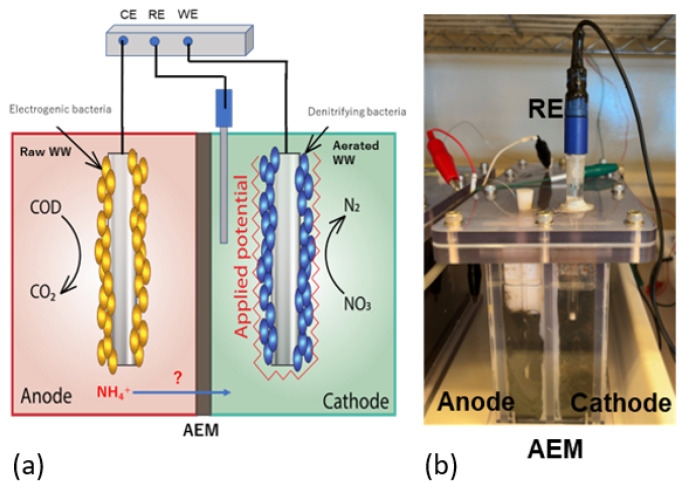
Two-chambered electrotrophic denitrification system. (**a**) Schematic diagram of the system. The anode chamber contains raw swine WW and the cathode chamber contains aerated WW. Two chambers are separated by AEM either produced in this study or commercial AMI-7001. (**b**) A photo of the reactor.

The selected AEM was tested for the efficiency of removal of organics from raw WW and nitrate from aerated WW using a 2 L, two-chambered biocathode BEDS (Figure 3); the reactors and procedures were described in our previous paper [11]. Briefly, carbon fiber (CFS-2; SO-EN Co., Ltd., Gunma, Japan) and a carbon brush with stainless steel core 800 K tips per 2.5 cm (Chieh Wang Industry and Trade Co, Hengshui, China) were used for the anode and cathode, respectively. The anode chamber was fed with raw swine WW containing organics and ammonium, and the cathode chamber was fed with aerated swine WW with low organics, but high nitrate, and phosphate. For the separator of the two chambers, either AMI-7001 or our AEM fabricated with 1c monomer (1 M monomer) and 2 mol% of CL was used. Nitrate removal in the cathode chamber and COD removal in the anode chamber were measured for both AEMs. This experiment was repeated in batch mode five times. 

### 2.7. Permselectivity Measurements

**Figure 4:**
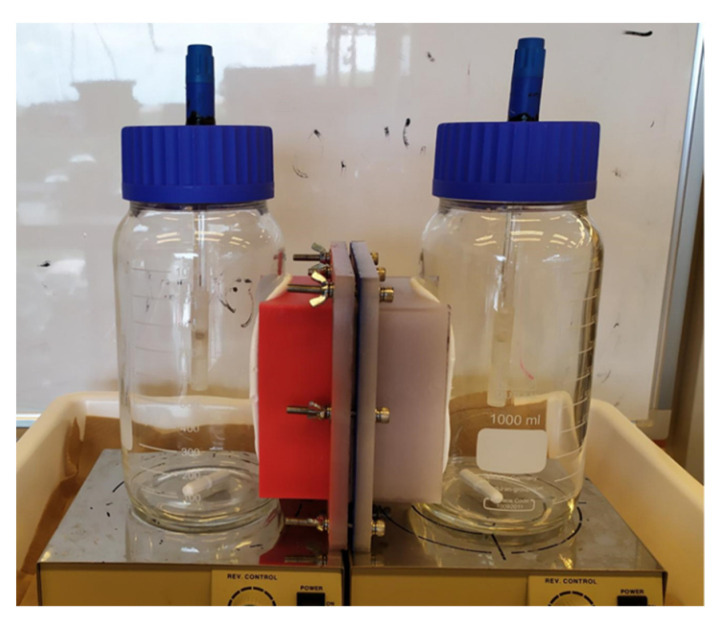
A photograph of the two-chambered device constructed for permselectivity measurement. Each chamber harbors a reference electrode. The chambers are separated by the AEM either produced in this study or commercial AMI-7001.

The ionic permselectivity was measured to understand the selectivity of the membrane towards the passage of counter-ion (cation). The two-chambered device for testing the permselectivity of the AEMs was constructed as shown in Figure 4, and measurements were conducted as described in the literature [12,13]. The volume of each chamber was 1 L. Plastic components were 3D printed (Ultimaker S5; Layertec BV, Zaltbommel, Netherlands) with polyethylene terephthalate glycol-modified printing material from the same company. The side of the reactor was sealed using 2 mm adhesive silicone foam (AS ONE, Osaka, Japan). Each chamber was filled with two different concentrations of KCl solution (C1 = 0.1 mol/L KCl, C2 = 0.5 mol/L KCl) [14] and stirred to ensure the solutions were well mixed. The membrane surface area for the experiment was 70 mm^2^. The electric potential difference between the silver chloride reference electrodes (Radiometer Analytical XR30; HACH Company) was measured after 1 h when ion movement had stabilized. 

## 3. Results and Discussion

### 3.1. Selection of the Best Monomer

From all tested monomers, only 1c provided strong gels at all CL concentrations (Table 1). Monomers 1a and 1b remained as viscous liquids or weak gels after polymerization using 2 and 4 mol% of the CL. With monomer 1d, problems were encountered with swelling of the second acrylamide monomer solution. This may have been due to the hydrophobic nature of the styrene moiety. Based on these results and because the 1c monomer was available as a solution that allows us to avoid working with the powder form of 1a and 1b monomers, we decided to use the 1c monomer for the membrane preparation. Another advantage of using the 1c monomer was the lowest price among tested monomers. 

### 3.2. Electrodialysis

**Figure 5:**
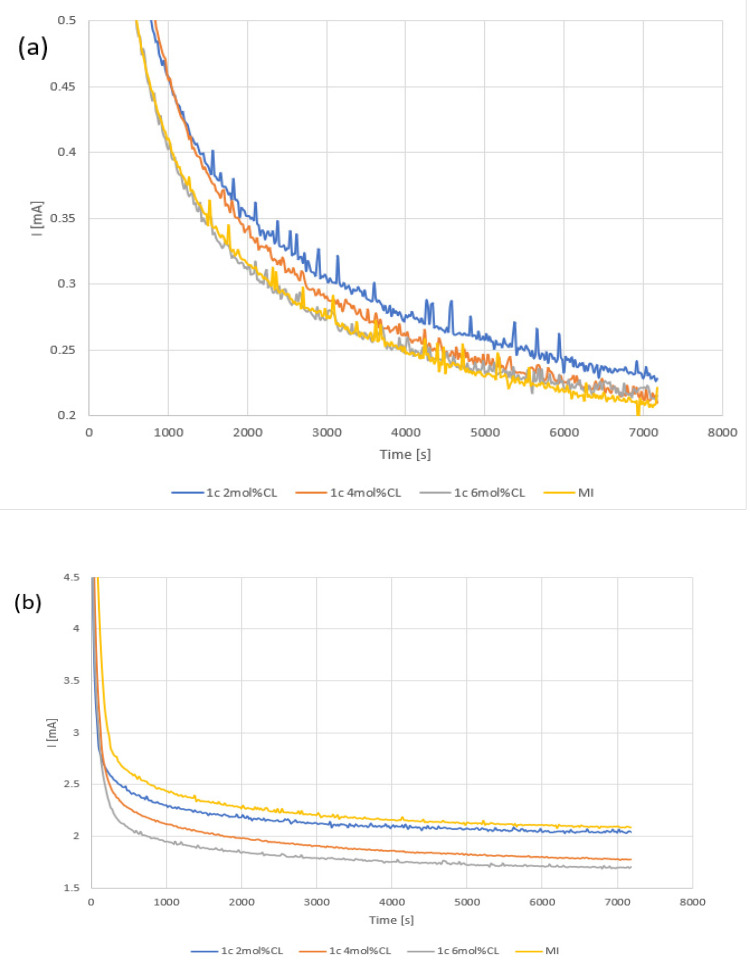
Chronoamperograms of electrodialysis experiments with 0.2 M phosphate buffer using AEM produced by the different cross linker concentrations. (**a**) Test at 1.0 V and (**b**) at 1.5 V. MI refers to AMI-7001 as a benchmark.

Phosphate removal was first tested with 200 mg/L phosphate buffer at 0.5, 1.0, and 1.5 V potentials. At 0.5 V, the measured potential currents were too low with high noise, making interpretation difficult. At 1.0 and 1.5 V, the current decreased with higher CL concentrations (Figure 5), indicating ion movement. Because we observe a trend of current decrease with higher CL concentration, further higher concentration of CL was not tested.

**Figure 6:**
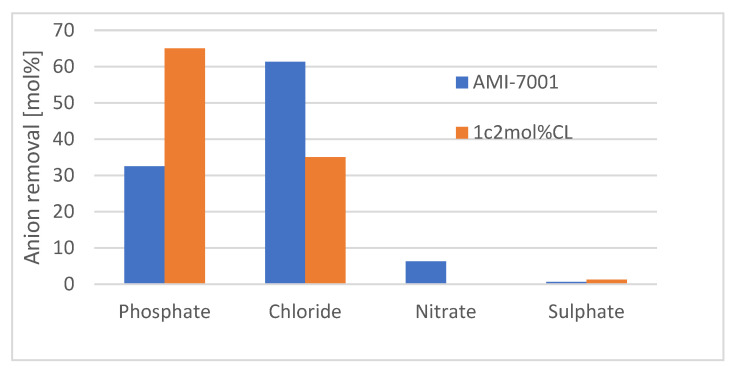
Selectivity of anion removal by the electrodialysis. Comparison of mol% removal of phosphate, chloride, sulphate, and nitrate ions from aerated swine WW, AMI-7001 (blue); 1c 2 mol%CL AEM (orange). AMI-7001 shows higher permeability to chloride anions and 1c 2 mol%CL shows higher permeability to phosphate anions.

Based on these results, the AEM prepared from monomer 1c and 2 mol%CL (hereafter called ‘1c 2 mol%CL AEM’) was used to remove phosphate from real aerated swine WW. Initial and final concentrations of phosphate in both chambers (− and + electrodes) and the cumulative charge were measured at 1.5 and 2 V potentials, and the efficiency of the removal process was calculated as necessary Ah to remove one gram of phosphate (Table 2). Our membrane showed a better phosphate removal rate and efficiency than the commercial one. To represent this difference in efficiency, the mol% of removed ions was calculated for the most abundant anions in the aerated swine WW (Figure 6). For AMI-7001, higher permeability to chloride anions than phosphate anions was observed. In contrast, our 1c 2 mol%CL AEM showed higher permeability to phosphate anions than chloride anions. AMI-7001 uses gel polystyrene cross linked with divinylbenzene whereas our membrane is Poly(dimethyldiallylammonium chloride) cross linked with *N,N*′-methylenebisacrylamide. The difference in charged polymer composition has an impact on the affinity to different ions [15,16] which may explain differences in anion permeability.

### 3.3. Electrotrophic Denitrification System

**Figure 7:**
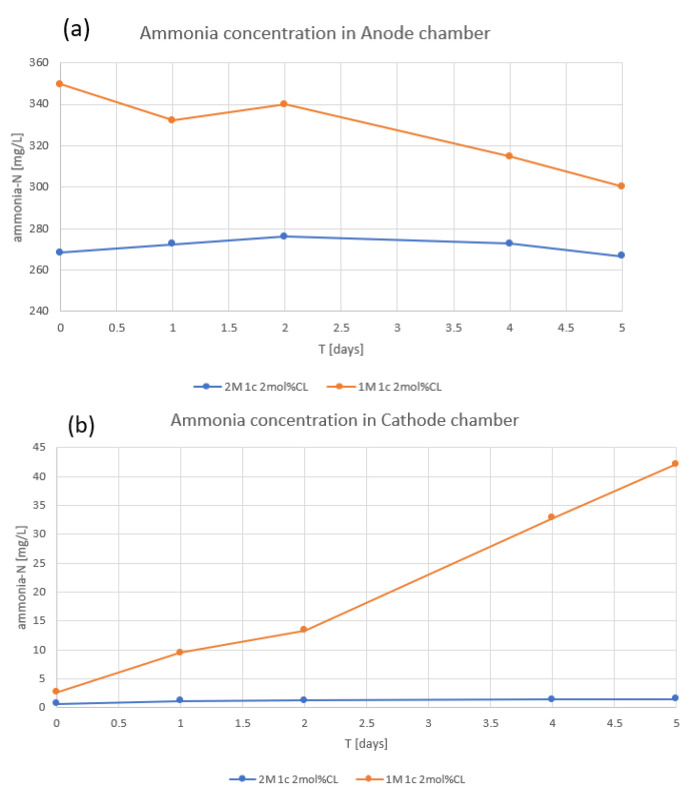
Ammonia flux from the anode to cathode chamber in the batch experiment using 1c 2 mol%CL AEM (orange) and the improved 2 M 1c 2 mol%CL AEM (blue). (**a**) ammonia concentration in the anode chamber. (**b**) ammonia concentration in the cathode chamber.

During the initial bioelectrochemical experiments using our 1c 2 mol%CL AEM, we observed ammonia leakage from the anode chamber to the cathode chamber (Figure 7). We tested the permselectivity of membranes and found 1c 2 mol%CL AEM to have low permselectivity (33%) compared with AMI-7001 (91%; Table 3). To improve the permselectivity of our AEM, we increased the concentration of the 1c monomer from 1 M to 2 M, which increased the permselectivity to 91%. Using this improved membrane, no flux of ammonia between chambers was observed (Figure 7).

**Figure 8:**
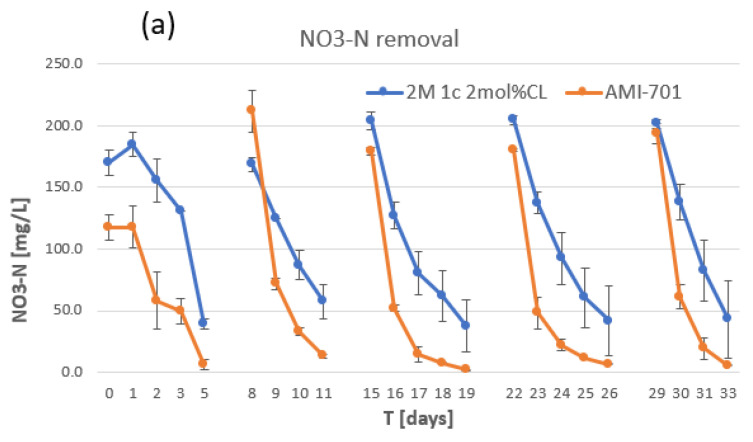

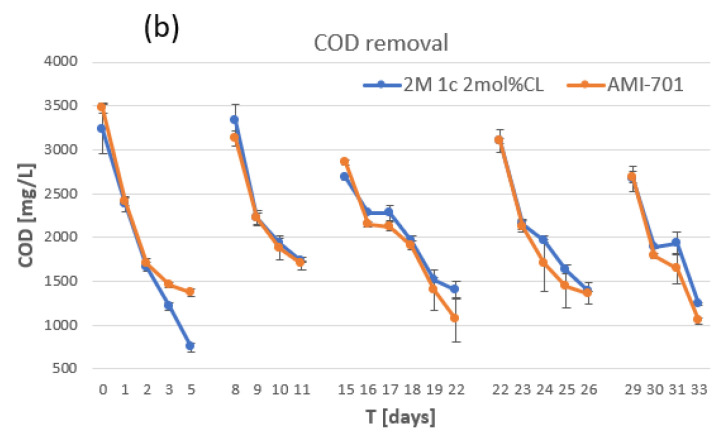
Nitrate and organic removal using electrotrophic denitrification system (**a**) Nitrate-nitrogen (NO_3_^−^-N) in cathode chamber and (**b**) organics (chemical oxygen demand) in anode chamber removal using our AEM 2 M 1c 2 mol%CL (blue) and AMI-7001 (red) through 5 batch modes.

Using the improved AEM (2 M 1c 2 mol%CL AEM), fiver batch modes of the bioelectrochemical denitrification experiment were conducted to compare the membrane’s efficiency with that of AMI-7001 in removing nitrate and organics (COD). WW samples from both chambers were analyzed daily (Figure 8). The removal of nitrate was faster with AMI-7001 than with our AEM, which indicates that WW treatment using our membrane would take longer to reach the nitrate discharge limit (100 mg/L nitrate-nitrogen [NO_3_-N] in Japan). No major difference was observed for COD removal from raw WW using the two membranes, which is an important part of swine WW treatment. 

### 3.4. Permselectivity

A 1 M solution of 1c monomer was used as starting concentration for AEM preparation. It works well for electrodialysis phosphate removal from swine WW. However, as stated, our original membrane (1c 2 mol%CL AEM) showed strong ammonia leakage between chambers of the BEDS and had a lower (33%) permselectivity than AMI-7001 (91%). High permselectivity of AEM for swine WW treatment in our electrotrophic denitrification system is crucial. It prevents ammonia leakage from the anode chamber to the cathode chamber which otherwise decreases our total nitrogen removal efficiency.

By increasing the monomer concentration to 2 M, the permselectivity of our AEM improved to a level comparable to that of AMI-7001 (Table 3). The measured permselectivity of AMI-7001 was close to the reported datasheet (90%) [17], with the slight difference possibly due to measurement errors or because the datasheet reported values in mol/kg rather than mol/L. The AEM with improved permselectivity (2 M 1c 2 mol%CL AEM) was used in our BEDS (Figure 8). A higher concentration of 1c monomer was not tested due to the high viscosity of 1c solution and consequent problem to fill pores of ceramic support. 

## 4. Conclusions

Our newly developed hydrogel AEM on a porous ceramic carrier showed very similar properties to the commercially available AMI-7001 membrane in the tested applications. Our membrane has a number of distinct advantages over AMI-7001: first, our AEM shows strong physical strength and is self-supporting owing to the ceramic carrier; second, the production method can easily be incorporated into any shape and size of porous ceramic on the market; third, the permselectivity of our AEM can be optimized by altering the charged monomer concentration. This simple modification of the fabrication method allows for customization for specific applications. These properties permit our proprietary AEM to be scaled up, which would be necessary for any practical usage, such as WW treatment. While further optimization of the process is required for this, no special equipment would be needed. Furthermore, because no organic solvents are used in the production process (water is the only solvent necessary), our AEM is relatively environmentally friendly. 

Another option to improve hydrogel membranes is the usage of porous plastics as carriers. Porous plastic is often used in WW treatment and is cheaper than ceramic. We plan to test plastics with low hydrophobicity, such as nylon, as carriers in the future. While porous polypropylene or polyethylene may not be suitable for our current fabrication process because of their high hydrophobicity, the addition of an organic solvent to the first monomer solution may overcome this issue.

A CEM produced using a similar method and a porous ceramic carrier has been successfully tested in microbial fuel cells and biosensors [18,19] in our laboratory, and we also plan to test our AEM in biosensor applications.

## 5. Patents

Fedorovich V.; Filonenko G.; Goryanin I; Schaffer-Harris G.K.; Simpson D.J.W.; Babiak P. Separator of a microbial fuel cell. WO 2019/160046 A1.

Kainuma M.; Prokhorova A.; Hiyane R.; Kazeoka M.; Goryanin I.; Babiak P. Concurrent raw and aerated wastewater treatment method using bioelectrochemical system. Japanese Patent Application Number: 2021-198900.

## Figures and Tables

**Table 1:** Results of the polymerization experiment with different crosslinker concentrations.

Monomer	Crosslinker (CL) [mol%]	Monomer Polymerization Gel Property	Acrylamide Swelling
1a	2	Viscous liquid	Not tested
	4	Weak gel	OK
	6	Strong gel	OK
1b	2	Viscous liquid	Not tested
	4	Viscous liquid	Not tested
	6	Strong gel	OK
1c	2	Strong gel	OK
	4	Strong gel	OK
	6	Strong gel	OK
1d	2	Liquid	Not tested
	4	Viscous liquid	Not tested
	6	Strong gel	No swelling

List of charged monomers used: 1a, [2-(acryloyloxy)ethyl]trimethylammonium; 1b, (3-acrylamidopropyl)trimethylammonium; 1c, diallyldimethylammonium; 1d, (vinylbenzyl)trimethylammonium. All monomers have Cl^−^ as the counterion.

**Table 2:** Phosphate removal from aerated swine WW by electrodialysis.

Voltage	Membrane	Initial Phosphate [mg/L]	Final Phosphate [mg/L] -Electrode	Final Phosphate [mg/L]	Cumulative Charge	Efficiency
1.5 V	AMI-7001	89.1	40.2	153	61.9	2.53
	1c 2 mol%CL	89.1	28.4	143	68.5	2.25
2 V	AMI-7001	86.2	15.3	179	121.5	3.44
	1c 2 mol%CL	86.2	3.2	156	132.6	3.19

**Table 3:** Permselectivity measurements using 0.1 M and 0.5 M KCl solutions.

Membrane	AMI-7001	1c 2 mol%CL	2 M 1c 2 mol%CL
Permselectivity	91%	33%	91%

## Data Availability

Not applicable.
